# Effect of a polyphenol-rich pomegranate extract on plasma trimethylamine N-oxide levels following an oral carnitine challenge: a randomized controlled crossover trial in healthy adults

**DOI:** 10.3389/fnut.2026.1822840

**Published:** 2026-05-20

**Authors:** Julia E. Haarhuis, Jennifer Ahn-Jarvis, George M. Savva, Priscilla E. Day-Walsh, Clare Ferns, Sarah Hughes, Silas Triller, Olla Al-Jaibaji, Mark Philo, Emrah Acaroz, Natalia Perez-Moral, Paul A. Kroon

**Affiliations:** 1 Quadram Institute Bioscience, Norwich Research Park, Norwich, United Kingdom; 2 Department of Obstetrics and Gynaecology, University of Cambridge, The Rosie Hospital, Cambridge, United Kingdom; 3 Department of Physiology, Development and Neuroscience, Loke Centre for Trophoblast Research, University of Cambridge, Cambridge, United Kingdom; 4 NIHR Norfolk Clinical Research Facility, Norwich Research Park, Norwich, United Kingdom

**Keywords:** bioavailability, ellagic acid, ellagitannins, pharmacokinetics, polyphenols, punicalagin, TMA

## Abstract

**Introduction:**

Polyphenol-rich pomegranate extract has been shown to inhibit microbial trimethylamine (TMA) production from L-carnitine. Previous clinical studies have examined effects of polyphenol-rich interventions on fasting trimethylamine N-oxide (TMAO) concentrations but have not assessed pharmacokinetic TMAO responses following an oral carnitine challenge (OCC). We investigated whether a single dose of pomegranate extract attenuates the plasma TMAO response to an OCC in healthy adults.

**Methods:**

This two-phase dietary intervention study enrolled 34 healthy, omnivorous adults. In Phase I, participants completed an OCC (1.5 g L-carnitine) to identify high TMAO producers (increase ≥ 5 μmol/L). Twenty high producers entered Phase II; an 18-day double-blind, randomized, placebo-controlled, crossover study with two 48-h pharmacokinetic interventions separated by a 10-day washout. Interventions consisted of an OCC with concurrent pomegranate extract (1.6 g) or placebo. Each OCC was preceded by a 48-h low-TMAO precursor run-in diet. Participants arrived fasted (>8 h), and all meals during the intervention periods were fully standardized to minimize dietary variability. Blood, urine, and stool samples were collected, and TMAO was quantified using LC-MS/MS. Differences in TMAO area under the curve (AUC) were analyzed using linear mixed-effects models.

**Results:**

Ninety one percent of participants meeting the Phase I inclusion criteria produced substantial TMAO quantities from L-carnitine. This proportion exceeds that of earlier reports. Pomegranate extract did not reduce TMAO AUC in the full Phase II cohort (placebo/pomegranate ratio 0.993, 95% CI 0.81–1.22; *P* = 0.945; *n* = 16). However, a *post hoc* subgroup analysis showed that the effect of the pomegranate extract on plasma TMAO differed by age and sex.

**Conclusion:**

Under tightly controlled dietary conditions, a single dose of pomegranate extract did not reduce post-OCC TMAO responses in the overall cohort. *Post hoc* analyses suggest potential sex- and age-dependent effects, warranting confirmation in larger, adequately powered studies.

**Clinical trial registration:**

https://clinicaltrials.gov/, identifier NCT06518343.

## Introduction

Cardiovascular disease (CVD) is a leading global cause of mortality, primarily driven by atherosclerosis (1, 2). Dietary modification is an important prevention strategy, as poor diet accounts for approximately 45% of cardiometabolic deaths in the United States and an estimated 10 million CVD deaths annually worldwide (3, 4). Among dietary factors, red meat consumption is strongly linked to increased CVD mortality risk (5–9), with elevated circulating trimethylamine N-oxide (TMAO) identified as a potential mechanistic pathway (10–12).

Dietary components shape gut microbiome composition and function by providing substrates such as fiber, fat, and protein (13). Through production of microbial metabolites, the gut microbiome influences host metabolism, immune function, and gut barrier integrity (14–16). Accumulating evidence indicates that the gut microbiome plays an important role in the pathogenesis of CVD (17–19). Several microbially derived metabolites have been associated with elevated CVD risk, including phenylacetylglutamine (20), imidazole propionate (21), and trimethylamine (TMA) (10, 22). Among these, TMA and its hepatically oxidized metabolite TMAO are the most extensively investigated in relation to cardiovascular health (15).

The gut microbiota produces TMA from dietary L-carnitine, choline, phosphatidylcholine, and betaine (23). These precursors are predominantly present in animal-based foods, including meat, fish, eggs, and dairy products, with betaine also abundant in plant sources such as spinach, beets, and whole grains (23). Red meat serves as a major dietary source of L-carnitine (23, 24). Initial evidence linking TMAO to CVD emerged from an untargeted metabolomics analysis in 2011 (10). Subsequently, a meta-analysis demonstrated that elevated TMAO concentrations were associated with a 74% increased risk of major adverse cardiovascular events (MACEs) among 39,314 participants with diabetes mellitus, CVD, or chronic kidney disease, and a 50% greater CVD risk among 22,945 participants (11).

*In vitro* experiments using colon models have shown that a polyphenol-rich pomegranate extract and the pomegranate polyphenol punicalagin can inhibit microbial TMA production from L-carnitine (25–27). However, human physiology involves complex processes of absorption, distribution, metabolism, and excretion that affect the bioavailability of dietary compounds (28). Punicalagin exhibits poor small intestinal bioavailability due to its large molecular weight (1084 Da) and extensive hydrogen bonding capacity (29), which enables it to reach the colon where interaction with gut microbiota occurs. This characteristic suggests that punicalagin may inhibit microbial TMA production, and subsequent hepatic TMAO formation, *in vivo*.

The bioavailability of L-carnitine from dietary sources ranges from 54% to 87%, whereas supplemental L-carnitine exhibits a much lower bioavailability, typically between 14% and 18% (30, 31). This difference occurs because supplemental L-carnitine absorption at higher doses relies primarily on passive diffusion across enterocyte membranes, whereas dietary L-carnitine utilizes both active and passive transport mechanisms (31). Unabsorbed L-carnitine reaches the colon where gut microbiota metabolize it to TMA. Substantial interindividual variation exists in TMA production, likely to reflect differences in gut microbiome composition and dietary patterns. Omnivores demonstrate a more than 20-fold greater TMAO responses to L-carnitine compared with vegetarians (32), and an oral carnitine challenge (OCC) revealed pronounced interindividual variation in plasma TMAO concentrations, particularly between omnivores and vegetarians (33). Higher dietary exposure to L-carnitine in omnivores, primarily from meat consumption, likely selects for gut microbiota with enhanced TMA-producing capacity, thereby elevating plasma TMAO concentrations. Notably, diets higher in red meat are associated with increased TMAO levels (34) and L-carnitine supplementation enhances gut microbial capacity for TMA production (35). These observations suggest that omnivorous individuals represent an appropriate study population for investigating interventions to reduce TMAO production.

Existing polyphenol interventions show that daily intake (over 4 to 8 weeks) of berry-, grape-, or apple-derived products can modestly lower fasting TMAO (36–39), yet none of these has examined how polyphenol-rich interventions influence full 48-h post-OCC plasma TMAO profiles from precursors such as L-carnitine. The assessment of plasma TMAO profiles after the exposure to a precursor, such as in an OCC, is essential to capture the effect of the polyphenols on the magnitude and timing of TMAO exposure rather than fasting TMAO concentrations alone. Moreover, only one prior study has evaluated an intervention derived from a source likely to contain ellagitannins (36). Given that our previously published *in vitro* experiments showed that punicalagin, an ellagitannin, can nearly abolish TMA formation (26, 27), ellagitannin-rich interventions represent promising candidates for further *in vivo* investigation targeting TMAO production.

This study addressed key knowledge gaps by characterizing 48-h post-OCC plasma TMAO pharmacokinetics in high TMAO-producing adults and testing whether a single dose of a polyphenol-rich pomegranate extract alters the dynamic TMAO response rather than fasting levels alone. Plasma AUC and pharmacokinetic parameters (C_*max*_, T_*max*_, t_1/2)_ for TMAO, L-carnitine, and γ-BB were assessed following OCC with pomegranate extract (1.6 g) or placebo. Secondary analyses included recovery of pomegranate metabolites in the urine, classification of TMAO producer phenotypes, associations with habitual dietary L-carnitine intake, and *post hoc* exploratory analyses of age- and sex-related differences in plasma TMAO AUC.

## Participants and methods

### Participants

**FIGURE 1:**
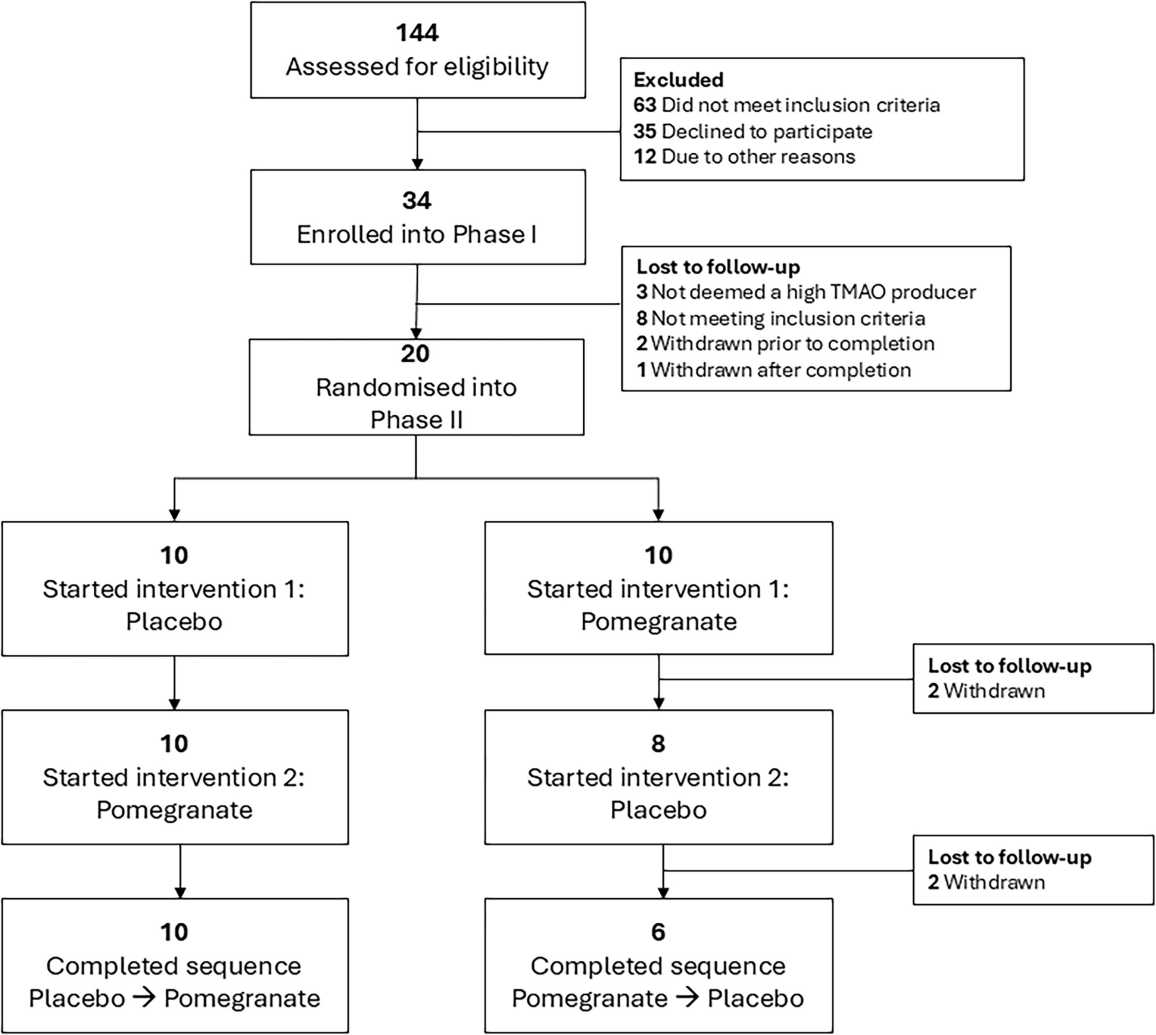
TESSA study CONSORT diagram.

The trial was conducted at the Quadram Institute Clinical Research Facility (CRF). A total of 34 healthy adults were enrolled in Phase I, with 32 completing the phase. Of those, 20 participants who were high TMAO producers and met all other inclusion criteria progressed to Phase II, with 16 completing the full study (Figure 1). Recruitment occurred from August 2024 to March 2025 through local media and social media platforms. Eligible participants for Phase I were healthy adults following an omnivorous diet (≥ 4 portions of meat weekly) with a body mass index (BMI) between 18.5–30 kg/m^2^ and residing within 40 miles of the Norwich Research Park. Omnivorous participants were recruited based on evidence demonstrating greater TMAO production from L-carnitine compared with vegetarians (32), thereby increasing likelihood of detecting treatment effects.

Exclusion criteria were: allergy or intolerance to standardized meal ingredients; tobacco use within 3 months of enrollment; bowel movement frequency < 5 times weekly; alcohol consumption > 14 units weekly; use of anticoagulant medications; abnormal blood pressure (≤ 90/60 or ≥ 160/100 mmHg); impaired kidney function (creatinine ≤ 59 or ≥ 104 μmol/L for males and ≤ 45 or ≥ 84 μmol/L for females; blood urea ≤ 2.5 or ≥ 7.8 mmol/L); medical conditions potentially affecting the primary outcome (e.g., diabetes, nonalcoholic fatty liver disease); dietary supplement use potentially affecting the primary outcome (e.g., fish oil, L-carnitine); pregnancy or lactation; active treatment for cancer or heart disease. All participants provided written informed consent before participation. This dietary intervention trial received ethical approval from the Quadram Institute Bioscience Human Research Governance Committee (QIB01/2024) and the South East Scotland Research Ethics Committee (24/SS/0047) and is registered at ClinicalTrials.gov (NCT06518343).

### Study design

Phase I consisted of two visits within 24 h during which participants consumed 1.5 g L-carnitine as an OCC. Fasting blood samples (after an > 8-h overnight fast) were collected immediately before and 24 h after L-carnitine administration. Participants completed a food frequency questionnaire (FFQ), and research nurses conducted medical assessments including height, weight, and blood pressure measurements. Plasma TMAO concentrations were analyzed in-house, while complete blood count, blood urea, creatinine, and estimated glomerular filtration rate (eGFR) were analyzed by Norfolk and Norwich University Hospital Pathology Department (Norwich, UK).

**FIGURE 2:**
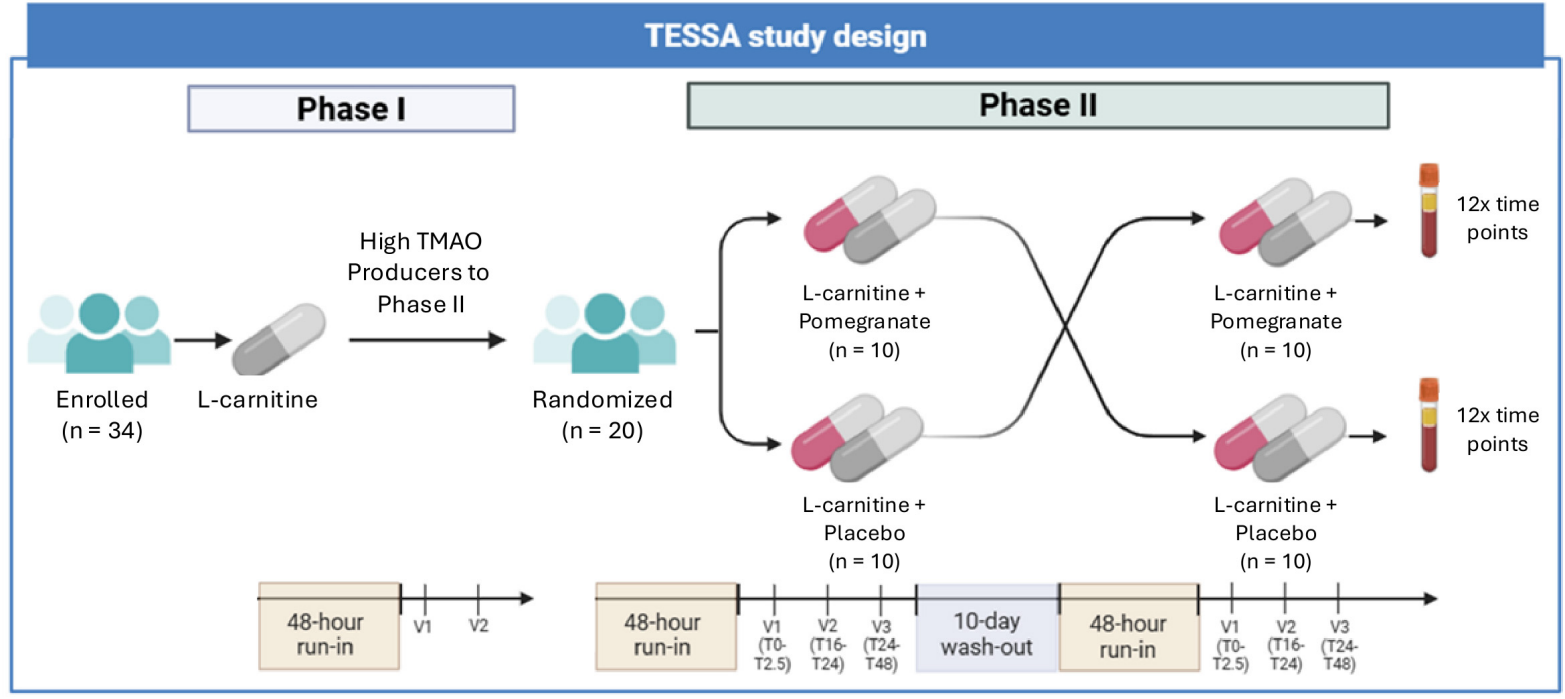
TESSA study design. The TESSA study was a two-phased study: Phase I consisted of two visits (V1, V2) and Phase II was an 18-day, double-blind, randomized, controlled crossover study consisting of two 48-h run-in periods and two 48-h pharmacokinetic studies with each three visits (V1-V3). The figure was created using BioRender.com.

Participants classified as high-TMAO producers (> 5 μmol/L and > 50% increase from baseline to 24 h) who met clinical eligibility criteria (normal kidney function and blood pressure) advanced to Phase II. Phase II was an 18-day, double-blind, randomized, placebo-controlled, crossover study comprising two interventions: an OCC with the pomegranate extract (1.6 g) and an OCC with the placebo. At Phase II enrollment, participants were randomly assigned to one of two intervention sequences (pomegranate followed by placebo, or placebo followed by pomegranate) (Figure 2).

Phase II encompassed six study visits over 18 ± 1 days. Each intervention included a 48-h run-in period followed by a 48-h pharmacokinetic assessment. A 10-day washout period separated the two interventions. Blood and urine samples were collected throughout each 48-h pharmacokinetic assessment. Blood was collected at 0, 0.5, 1, 1.5, 2, 2.5, 16, 18, 20, 22, 24, and 48 h post-OCC. Participants collected all their urine for 48 h in batches (0–2.5, 2.5–16, 16–18, 18–20, 20–22, 22–24, 24–48 h). Dietary intake during study days was fully controlled and standardized (see section “Run-in diet and standardized meals”).

Harms and unintended effects were assessed systematically by a research nurse at the start of each study visit using a questionnaire. No serious adverse events were reported.

Patient and public involvement (PPI) was undertaken during study preparation to assess the clarity and acceptability of participant-facing materials. Three members of a PPI group reviewed the Participant Information Sheet (PIS) and the sheet specifying the run-in diet and standardized meals. The PPI group provided structured feedback via an online survey. Feedback focused on readability, understanding of study procedures, and perceived feasibility of dietary adherence. Based on this feedback, clarifications were made to the PIS regarding TMAO producer status and eligibility for Phase II of the study. No further substantive concerns were raised.

### Randomization and blinding

Participants entering Phase II were randomized to one of two intervention sequences (pomegranate followed by placebo, or placebo followed by pomegranate) in a single block of 20. The randomization sequence was computer-generated by a statistician, and allocation concealment was maintained by an independent researcher. Study scientists remained blinded to treatment allocation until data analysis completion.

### Preparation of pomegranate extract and placebo capsules

Pomegranate extract (Dermogranate^®^, Medinutrex, Catania, Italy) and placebo capsules were prepared in the Quadram Institute Clinical Research Facility research kitchen using a Profiller 1100 (Torpac Inc., Fairfield, NJ, USA). Placebo capsules comprised microcrystalline cellulose (REDWELLS HEALTH LTD, London, UK) combined with 10% (w/w) gum Arabic (CAS 9000-01-5, Willy Benecke, Hamburg, Germany), matching the gum Arabic content of the pomegranate extract, and 1% (w/w) brown food coloring (rainbow dust edible powder color, Cake Decorating Co., UK) to ensure that the pomegranate and placebo capsules were identical in appearance. DRcaps^®^ size 0 capsules (Capsugel, Lonza, Slough, UK) were loaded into the filling device, and pomegranate extract or placebo contents were dispensed, spread using a powder spreader, and sealed.

Both placebo and pomegranate capsules were acid-resistant with delayed-release properties, enabling intestinal rather than gastric release. Capsules were packaged in 28 mL pill bottles, each containing four pomegranate capsules (400 mg each) or four placebo capsules, along with 3 × 500 mg L-carnitine (Carnipure^®^) capsules (VitaminExpress LLC, Irvine, CA, USA) and a silica gel sachet. Capsules underwent microbiological safety testing at ALS Laboratories (UK) before use.

### Run-in diet and standardized meals

To minimize TMAO production from sources other than the OCC, participants followed a 48-h low-carnitine (and low-ellagitannin) run-in diet before Phase I and II visits (Figure 2), using a list of foods to avoid during the run-in period. During each of the Phase II interventions, participants only consumed provided, standardized meals with limited L-carnitine, choline, and ellagitannins (≤ 200 mg/day for choline and L-carnitine), based on United States Department of Agriculture FoodData Central and published literature (23, 40–43). To ensure compliance to the standardized meal plans, participants’ dietary intake during the Phase II interventions were tracked using dietary records.

To validate the 48-h run-in diet, a coefficient of variation (CV) in baseline plasma TMAO was calculated across three fasting measurements (Phase I baseline, Phase II intervention 1 baseline, and Phase II intervention 2 baseline). More than half of the participants exhibited a CV below 50% (Supplementary Figures 6A,B). In comparison, the BERI study (NCT03213288), which measured plasma TMAO variability across six time points without dietary restrictions for trimethylamine precursors, reported that most participants exceeded 50% CV (44). These findings demonstrate that the 48-h run-in diet effectively maintained baseline plasma TMAO within an acceptable range (0–5 μmol/L) for most participants. Outliers with high fasting plasma TMAO levels may indicate lack of dietary adherence.

During the Phase II study visits, participants consumed only standardized meals provided directly after blood draws at time points 0, 2.5, 16, 18, 20, and 24 h, as well as standardized meals taken home between study visits 2 and 3 of each pharmacokinetic intervention. The standardized meals ensured that participants followed a diet that was low in TMAO-precursor nutrients and ellagitannins.

Caloric needs were calculated based on each participant’s activity level, sex, age, and BMI, and meal plans were assigned accordingly (ranging from 2,000–3,000 kcal/day). Dietary intake was recorded using Nutritics (version 6.20). If meals were not fully consumed, leftovers were weighed, and the identical remaining amount was provided during the second pharmacokinetic intervention, ensuring that each participant’s meals were consistent across intervention periods. Total daily energy and macronutrient intake did not differ significantly between the placebo and pomegranate interventions.

### Food frequency questionnaire (FFQ)

Participants completed an online, graphical FFQ (VioScreen™, Viocare Inc., Princeton, NJ, USA) assessing dietary intake over the 30 days preceding Phase I to estimate habitual L-carnitine consumption. This FFQ was validated in the United States and the questions represent Western dietary patterns (45). Total L-carnitine intake was calculated by multiplying published L-carnitine content values (mg/g) for food items (23, 40, 41) by the quantity (g) of L-carnitine-containing foods reported in the FFQ.

### Chemicals

Water was purified to 18 MΩ⋅cm using a Milli-Q system. Fisher Scientific Limited (Loughborough, UK) supplied high-performance liquid chromatography (HPLC) grade solvents and other reagents. Trimethylamine hydrochloride (CAS 593-81-7), TMAO (CAS 1184-78-7), γ-butyrobetaine [γ-BB, commercially available as 3-(Carboxypropyl)trimethylammonium chloride, CAS 6249-56-5], urolithin A (CAS 1143-70-0), urolithin B (CAS 1139-83-9), ellagic acid (CAS 476-66-4), trichloroacetic acid, dimethyl sulfoxide, glacial acetic acid, sulfatase (CAS 9016-17-5), β-glucuronidase (CAS 9001-45-0), and heptafluorobutyric acid were purchased from Merck (Darmstadt, Germany). L-carnitine (CAS 541-15-1) and ammonium acetate were obtained from Fisher Scientific Limited. Isotopically labeled internal standards included L-carnitine-(trimethyl-d9) (CAS 126827-79-0, Cambridge Isotope Laboratories, Tewksbury, MA, USA), trimethylamine-d9 hydrochloride (CAS 18856-86-5, LGC Standards, Teddington, UK), trimethylamine-d9 N-oxide (CAS 1161070-49-0, LGC Standards), and γ-butyrobetaine-d9 (CAS 479677-53-7, Santa Cruz Biotechnology, Dallas, TX, USA). Punicalagin (CAS 65995-63-3) and punicalin (CAS 65995-64-4) were obtained from BOC Science (Shirley, NY, USA) and Apollo Scientific (Stockport, UK), respectively.

### Collection and processing of biological samples

Venous blood was collected into ethylenediamine tetra-acetic acid (EDTA) tubes for plasma isolation and serum separator tubes for serum collection. EDTA tubes were immediately centrifuged at 3,000 × *g* for 15 min. Plasma samples were aliquoted (5–6 aliquots of 350 μL) and stored at −80 °C until batch analysis by liquid chromatography-tandem mass spectrometry (LC-MS/MS). Serum separator tubes were analyzed by Norfolk and Norwich University Hospital Pathology Department for eGFR, creatinine, and urea.

48-h urine was collected in urine pre-weighed containers with boric acid (0.5 g/L) as a preservative. Total urine volumes were obtained by dividing the weight of the urine by the specific gravity, measured using a One Step specific gravity dip stick. Urine was aliquoted into 5 tubes of 1 mL and stored at −80 °C until batch analysis.

### LC-MS/MS analysis of L-carnitine metabolites in blood plasma

Phase I plasma samples were analyzed for TMAO within two weeks of collection using the standard addition method. Plasma samples were split into five aliquots, and known TMAO concentrations (0–20 μmol/L) were added to each aliquot. Then, 50 μL of 0.2 mol/L acetic acid containing d9-TMAO was added, followed by 150 μL ice-cold trichloroacetic acid, and samples were incubated at 4 °C for 10 min to precipitate proteins. Aliquots were centrifuged at 13,000 rpm for 10 min at 4 °C, and 150 μL supernatant was transferred to Chromacol vials containing 100 μL water for dilution.

Phase II blood plasma samples were analyzed in batch following study completion. Analysis of TMAO, L-carnitine, γ-BB, and TMA in Phase II samples followed previously described methods (25, 46). To account for matrix effects in the LC–MS/MS analysis, a pooled plasma matrix was prepared by combining equal volumes of baseline plasma samples from multiple study participants prior to calibration curve construction and quality control sample preparation. Baseline analyte concentrations in pooled samples were determined by standard addition method, and standard curve concentrations were adjusted accordingly.

A targeted analysis was performed using isotopically labeled internal standards for L-carnitine, γ-BB, TMA, and TMAO. An Agilent 1290 Infinity II LC System coupled to a 6490 Triple Quad LC-MS (Agilent Technologies, Santa Clara, CA, USA) was used. Metabolites were separated using an Acquity UPLC BEH C8 1.7 μm column (Waters, Milford, MA, USA) with mobile phase comprising 10 mmol/L ammonium acetate and 0.05% heptafluorobutyric acid in Milli-Q water (solution A) and 10 mmol/L ammonium acetate and 0.05% heptafluorobutyric acid (dissolved in 50 mL Milli-Q water) in methanol (solution B).

### LC-MS/MS analysis of polyphenols and urolithins in urine

Pomegranate polyphenols (punicalagin, punicalin, ellagic acid) and urolithins were quantified in urine by LC-MS/MS (Waters TQ Absolute, Wilmslow, UK). Standard curves were prepared in pooled baseline urine from multiple participants collected before intervention, with concentrations ranging from 0 to 16 μg/mL for each compound. Urine samples and standards were transferred to 96-well plates, taxifolin (1 μg/mL) was added as internal standard, and samples were vortexed and centrifuged at maximum speed for 2 min before LC-MS/MS analysis.

Mobile phase consisted of 0.1% formic acid in distilled water (solvent A) and 0.1% formic acid in acetonitrile (solvent B). One microliter of each sample was injected onto a Waters HSS T3 column (100 × 2.1 mm; 1.8 μm particle size) at 35 °C with 0.4 mL/min flow rate. The gradient profile was: 3% solvent B for 5 min, increased to 20% over 4 min, further increased to 50% over 2 min, then to 95% over 1 min, followed by re-equilibration to 3% for 2 min (total run time 14 min). Polyphenols were identified by matching retention time and m/z ([M-H]^–^) with authentic standards. Peak assignment and quantification were performed using SYNAPT G2-Si software (Waters).

Because urinary urolithins are predominantly present as conjugates, concentrations were quantified using aglycone standards and corrected using experimentally derived relative response factors. Details of relative response factor determination are provided in the Supplementary methods.

### Sample size calculation and statistical analyses

#### Sample size

*In vitro* experiments demonstrated that the pomegranate extract reduced TMA production at 48 h by 48.2–76.4%, depending on the dose (25, 26). Human intervention studies showed that the daily consumption of polyphenol-rich grape extract for four or eight weeks reduced fasting plasma TMAO concentrations by 63.6% and 78.6%, respectively, compared with baseline (37, 38). However, no published studies have examined whether polyphenol-rich extracts reduce TMAO production following an OCC, limiting available data for effect size estimation. A within-person standard deviation of 1.178 μmol/L (log-transformed) for maximum TMAO concentration after a 1.5 g OCC was derived from a study by Wu et al. (33). Based on this variability, 16 participants would provide 80% power to detect a 60% reduction in TMAO production at α = 0.05. Twenty participants were randomized to Phase II to account for potential withdrawals.

#### Data analyses

Data were analyzed per protocol. For Phase I, all participants who completed the OCC were included in analyses relevant for that phase (*n* = 32). For Phase II, the participants who completed both intervention periods were included in analyses relevant for that phase (*n* = 16), unless otherwise stated. Statistical significance was defined as *P* < 0.05.

Pharmacokinetic effects of pomegranate extract on plasma TMAO were estimated by comparing log-transformed AUC (log AUC) between pomegranate extract and placebo interventions. A linear mixed model with intervention as fixed effect and participant as random effect was used to estimate TMAO AUC difference between treatments. For clarity, AUC differences are displayed on a linear scale (μmol/L × h) in figures, while statistical interpretations are based on log-transformed models. AUC was calculated using the trapezoidal rule via the trapz() function from “pracma” package (47), from time 0 to the last measured time point (AUC0-t) without extrapolation to infinity or adjustment to baseline concentrations. Where fewer than three data points were missing, curves were estimated using imputed values. One participant presented abnormal baseline TMAO values (> 45 μmol/L) in one intervention at three time points, which returned to baseline by 1.5 h post-challenge. Therefore, to better capture TMAO pharmacokinetics in response to the OCC, the first three TMAO concentration data points were excluded for this participant.

Additional pharmacokinetic parameters (C_*max*_, T_*max*_, t_1/2_) were calculated from plasma TMAO and L-carnitine concentrations using R package “pracma” (47). C_*max*_ and T_*max*_ were determined as the highest observed TMAO concentration and corresponding time point, respectively, per participant and intervention. TMAO t_1/2_ was estimated by fitting linear regression to log-transformed concentrations over the final three time points of each curve, corresponding to the terminal elimination phase, following standard log-linear pharmacokinetic modeling methods.

*Post hoc* analyses examined whether intervention effects on plasma TMAO differed by age and sex. Age groups were defined using a median split. To test whether the intervention effect differed by age group, a linear mixed model was fitted with TMAO log AUC as the outcome, fixed effects for intervention (placebo vs. pomegranate), age group, and their interaction (intervention × age group), and a random intercept for participant. To further assess combined age-sex effects, differences in TMAO log AUC (placebo minus pomegranate) were calculated per participant and analyzed using linear models including age group, sex, and their interaction. The linear mixed model uses all individual measurements and accounts for repeated measures, while the model assessing differences in TMAO log AUC summarizes each participant’s response for direct comparisons between age-sex subgroups. When significant interaction effects were observed, *post hoc* pairwise comparisons between age-sex subgroups were conducted using estimated marginal means with Tukey adjustment for multiple comparisons.

Graphs were generated using GraphPad Prism version 10.4.2 (GraphPad Software, Boston, MA, USA) and R version 4.4.1 [R Core Team, 2024] in RStudio (Posit Software, PBC, Boston, MA, USA). Statistical analyses were performed in RStudio using packages with “ggpubr” (48), “FSA” (49), “lmerTest” (50), “emmeans” (51), and base functions from the “stats” package.

## Results

### Phase I results

#### Study enrollment and participant flow

Of the 144 individuals who expressed interest, 34 participants enrolled in Phase I (Figure 1). Thirty-two participants completed Phase I, of whom 29 (90.6%) were classified as high-TMAO producers (≥ 5 μmol/L and ≥ 50% increase from baseline to 24-h follow-up). Twenty participants were randomized to Phase II. Four participants withdrew from Phase II: two completed only one intervention and two withdrew at study initiation. Sixteen participants completed both Phase II interventions and were included in the complete analysis. The trial ended as planned after completion of recruitment and follow-up for all participants.

#### Phase I participant characteristics

Among the 32 participants who completed Phase I, 18 (56.3%) were male and 14 (43.8%) were female (Table 1). Mean age was similar between males and females (41.2 ± 14.1 vs. 40.1 ± 14.2 years).

**TABLE 1:** Baseline characteristics of the participants who completed Phase I.

Characteristic	All participants (*n* = 32)	Male (*n* = 18)	Female (*n* = 14)
Age, years (mean ± SD)	40.7 ± 14.2	41.2 ± 14.1	40.1 ± 14.9
Height, cm (mean ± SD)	171.5 ± 8.9	176.4 ± 7.3	165.0 ± 6.3
Weight, kg (mean ± SD)	75.8 ± 11.9	80.3 ± 10.4	70.1 ± 11.5
BMI, kg/m^2^ (mean ± SD)	25.8 ± 3.2	25.8 ± 2.7	25.8 ± 3.9
Baseline TMAO, μ mol/L (mean ± SD)	4.4 ± 4.2	4.4 ± 4.0	4.5 ± 4.6
24-h TMAO, μ mol/L (mean ± SD)	41.6 ± 28.9	41.0 ± 25.7	42.5 ± 33.6
TMAO increase, μ mol/L (mean ± SD)^1^	37.1 ± 28.5	36.6 ± 25.2	37.9 ± 33.2

^1^Calculated as 24-h TMAO minus baseline TMAO.

#### Plasma TMAO response to the OCC in Phase I

**FIGURE 3:**
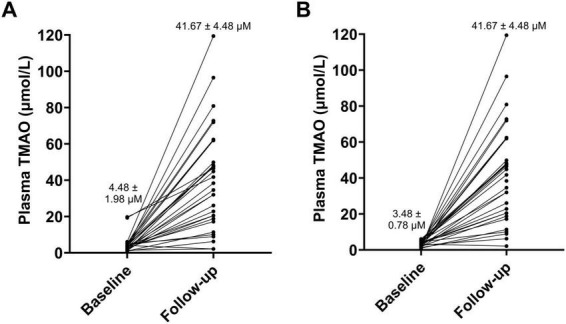
Individual trimethylamine N-oxide (TMAO) production after the oral carnitine challenge (OCC) in Phase I. Individual production of TMAO **(A)** for all participants who completed Phase I (*n* = 32) and **(B)** after excluding outliers at baseline (*n* = 29) using the interquartile range method (IQR) (69). Presented values are mean ± SD. Fasted blood samples were collected from TESSA participants prior to and 24 h after the OCC. Whole blood samples were centrifuged to obtain plasma and stored at –80 °C until LC-MS/MS quantification using a d9-TMAO internal standard.

Fasting plasma TMAO concentrations (±SD) measured before the OCC averaged 4.48 ± 4.19 μmol/L. Two participants exceeded the upper interquartile range outlier threshold (> 7.3 μmol/L). After excluding these outliers, mean baseline TMAO was 3.48 ± 1.46 μmol/L (*n* = 30) (Figure 3).

Following the OCC, 29 of 32 participants (90.6%) exhibited plasma TMAO exceeding 5 μmol/L, with a mean (±SD) increase of 40.0 ± 26.2 μmol/L. To compare the prevalence of high-TMAO producers with other cohorts, we applied the classification criteria from Wu et al. (33) which defined high-TMAO producers as those exceeding 10 μmol/L plasma TMAO at 24 h post-challenge. Using these criteria, 28 of 32 participants (87.5%) qualified as high-TMAO producers (Supplementary Figure 7).

#### Demographic and dietary associations with TMAO response

**FIGURE 4:**
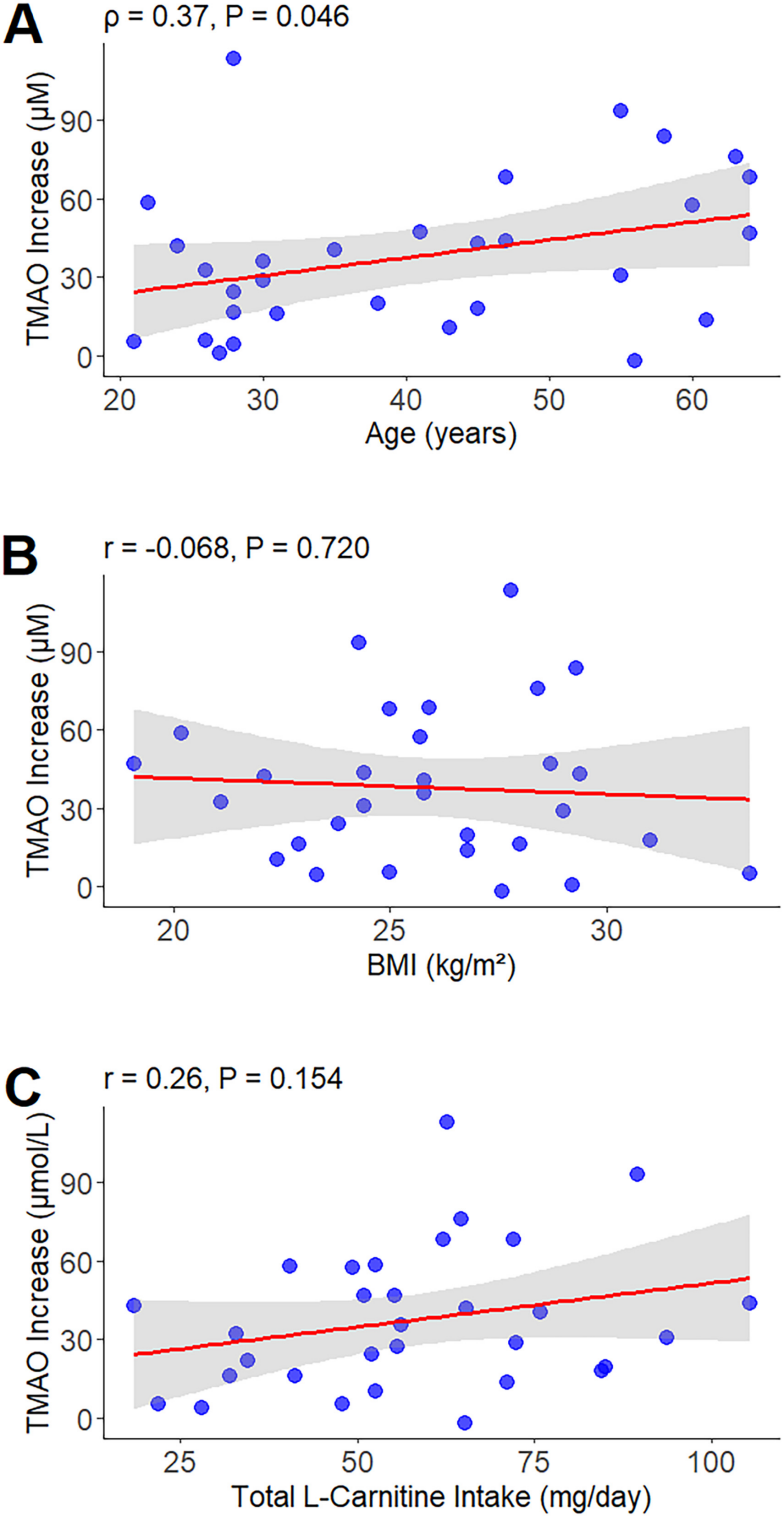
Correlations between the trimethylamine N-oxide (TMAO) response and **(A)** age, **(B)** body mass index (BMI), and **(C)** habitual L-carnitine intake. BMI, age, and L-carnitine intake were measured as secondary outcomes during Phase I. TMAO response is defined as the change in plasma TMAO concentration from baseline to 24 h following the oral carnitine challenge (OCC). Each point represents an individual participant (*n* = 31–32). Associations were evaluated using a Spearman correlation for age and a Pearson’s correlation for BMI and habitual L-carnitine intake. One outlier for L-carnitine intake was identified using the interquartile range method (IQR) (69) and excluded from the analysis.

Age was significantly correlated with TMAO response in Phase I (Figure 4A; ρ = 0.37, *P* = 0.046), with older participants demonstrating greater TMAO increases from baseline to 24 h. A rank-based Theil-Sen regression showed a slope of 0.99 μmol/L per one-year increase in age (*P* = 0.001), consistent with the correlation analysis.

No significant correlation was observed between BMI and TMAO response (Figure 4B; *r* = 0.28, *P* = 0.133). However, a trend was observed between habitual intake of dietary L-carnitine and the increase in TMAO from baseline to 24 h (Figure 4C; *r* = 0.26, *P* = 0.154), although this was not statistically significant. A linear regression showed a slope (β ± SEM) of 0.33 ± 0.23 μmol/L per mg of L-carnitine [*t*(29) = 1.46, *P* = 0.154].

### Phase II results

#### Phase II participant characteristics

Baseline characteristics for the 16 participants from Phase I who completed Phase II are shown in Table 2. Ten (62.5%) were male and 6 (37.5%) were female.

**TABLE 2:** Baseline characteristics, measured in Phase I, of the participants who completed Phase I and Phase II.

Characteristic	All participants (*n* = 16)	Male (*n* = 10)	Female (*n* = 6)
Age, years (mean ± SD)	40.1 ± 13.4	39.6 ± 13.4	40.8 ± 14.7
Height, cm (mean ± SD)	172.5 ± 8.8	177.1 ± 7.7	165.0 ± 4.5
Weight, kg (mean ± SD)	75.7 ± 11.4	80.5 ± 10.4	67.8 ± 8.8
BMI, kg/m^2^ (mean ± SD)	25.4 ± 2.8	25.7 ± 2.7	24.9 ± 3.0
Baseline **TMA**O, μmol/L (mean ± SD)	4.3 ± 4.2	4.8 ± 5.2	3.3 ± 1.4
24-h **TMA**O, μmol/L (mean ± SD)	44.6 ± 21.9	38.7 ± 19.2	54.3 ± 24.3
**TMA**O increase, μmol/L (mean ± SD)^1^	40.3 ± 21.2	33.9 ± 18.1	51.0 ± 23.1

^1^Calculated as 24-h TMAO minus baseline TMAO, measured in Phase I.

#### Effect of pomegranate extract on plasma TMAO response

**FIGURE 5:**
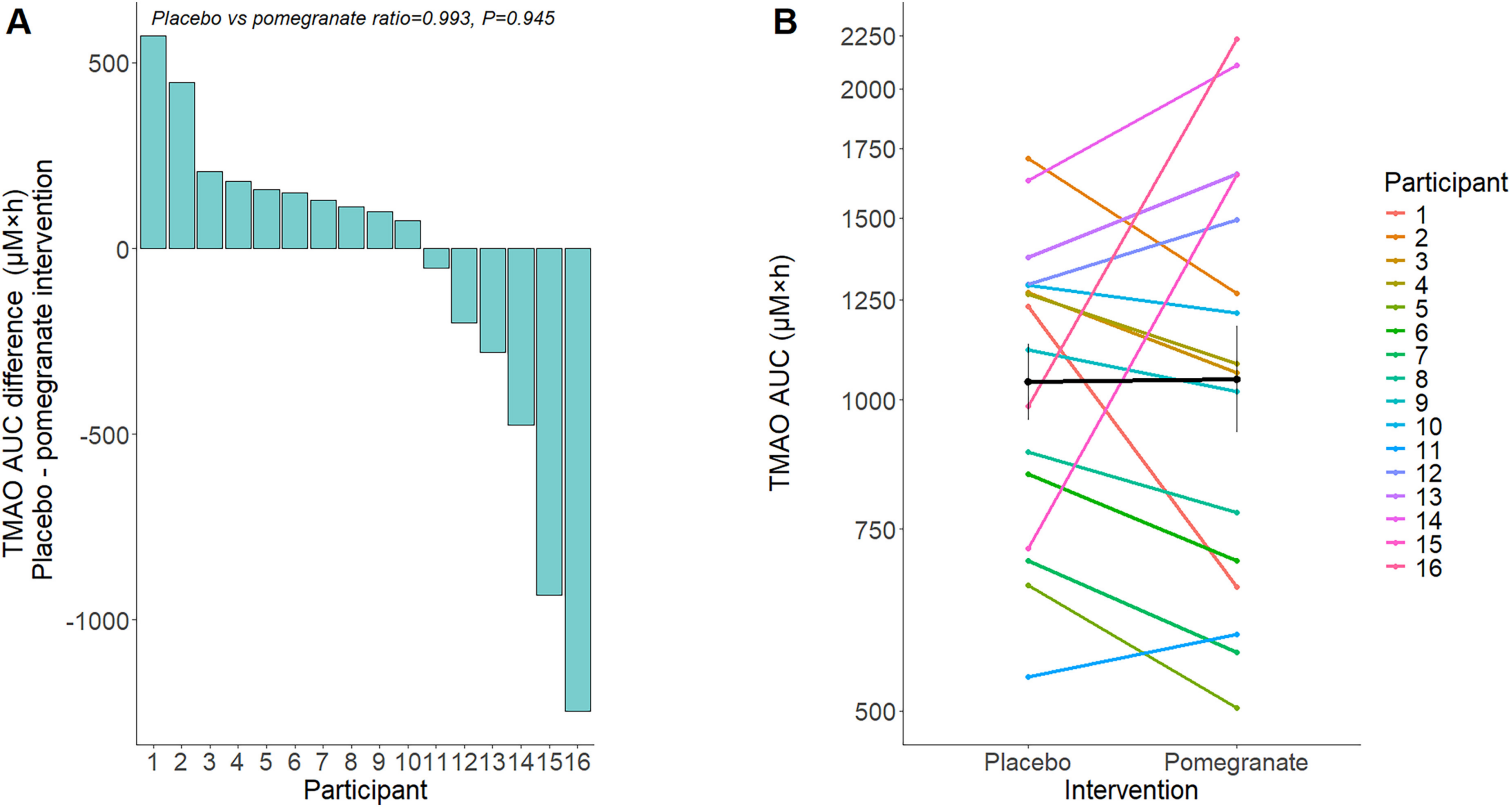
Individual trimethylamine N-oxide area under the curve (TMAO AUC) difference between the placebo versus the pomegranate extract. Participants (*n* = 16) underwent two oral carnitine challenges (OCCs), consuming 1.5 g L-carnitine once with a placebo and once with a pomegranate extract. For 48 h after each OCC blood plasma was collected to estimate the TMAO AUC. Graphs show **(A)** the difference between TMAO AUC after the placebo versus the pomegranate intervention, with positive values indicating that the pomegranate intervention reduced the TMAO AUC compared to the placebo and **(B)** paired TMAO AUC comparisons between the interventions, with the black line showing the mean. In figure **(A)**, AUC values are displayed on the linear scale, while statistical interpretations are based on the log-transformed model.

Ten of 16 participants exhibited lower TMAO AUC following pomegranate intervention compared with placebo (Figure 5). No significant mean difference was observed in TMAO log AUC between interventions (β = 0.0067, SE = 0.096, *P* = 0.945). Estimated mean TMAO AUC, based on the linear mixed model, was 1,041 μmol/L × h for placebo and 1,048 μmol/L × h for pomegranate, with a (placebo/pomegranate) ratio of 0.993 (95% CI: 0.809, 1.22). These results indicate that a single pomegranate extract dose did not significantly affect plasma TMAO concentrations following the OCC. Individual TMAO trajectories are shown in Supplementary Figure 9.

#### Age- and sex-dependent effects of pomegranate extract on TMAO response

**FIGURE 6:**
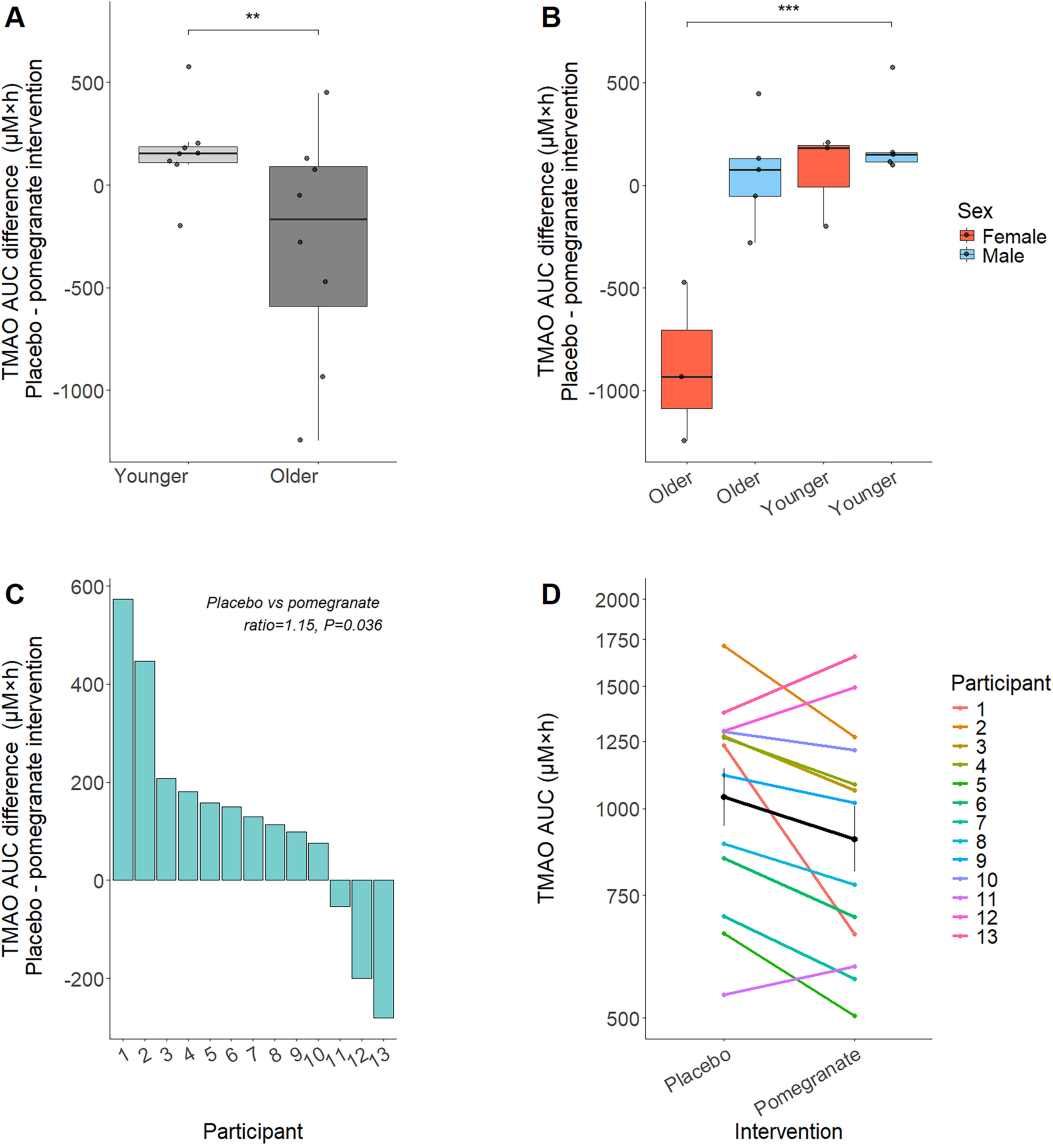
Distribution of trimethylamine N-oxide area under the curve (TMAO AUC) difference between placebo versus pomegranate intervention by sex and age group **(A,B)**, and difference between the placebo versus the pomegranate extract for female participants in the “Younger” group and all male participants (both “Younger” and “Older” groups) **(C,D)**. Graphs showing **(A)** TMAO AUC by age group, defined by a median split at 36.5 years (*n* = 8 “Younger”, *n* = 8 “Older”), **(B)** TMAO AUC by age group and sex, **(C)** the difference between TMAO AUC after the placebo versus the pomegranate intervention (*n* = 13), with positive values indicating that the pomegranate intervention reduced the TMAO AUC compared to the placebo and **(D)** paired TMAO AUC comparisons between the interventions, with the black line showing the mean. In graph **(C)**, AUC values are displayed on the linear scale, while statistical interpretations are based on the log-transformed model. Participants (*n* = 13–16) underwent two oral carnitine challenges (OCCs), consuming 1.5 g L-carnitine once with a placebo and once with a pomegranate extract. For 48 h after the OCC blood plasma was collected to estimate the TMAO AUC. **P < 0.005, ***P < 0.001.

Although the overall effect of the pomegranate extract on plasma TMAO AUC was null (*P* = 0.945), we observed that the participants with increasing TMAO were older (Figure 6A), and in particular the three oldest women (*n* = 3; 53.3 ± 5.7 years) of the cohort (Figure 6B). A *post hoc* linear mixed model with TMAO log AUC as outcome and fixed effects for intervention (placebo vs. pomegranate), age group, and their interaction revealed a significant intervention × age group interaction (*P* = 0.037), indicating that the effects of the intervention differed by age. A further analysis of individual placebo vs. pomegranate log AUC differences, using a linear model with age group, sex, and their interaction as predictors, revealed a significant age × sex interaction on the intervention effect (*P* = 0.004), indicating that the identified difference by age varied with participant sex. However, this is a *post hoc* analysis with very small numbers in each age/sex subgroup (*n* = 3 for each of the younger and older female group; *n* = 5 for each of the younger and older male group).

These observations align with a cross-sectional study in 51 adults with obesity suggesting altered TMAO metabolism in postmenopausal women (52). In light of this, in combination with the observed age × sex interaction, we conducted a *post hoc* analysis excluding the three female participants in the “Older” group, which are of postmenopausal age based on reports by the WHO (53) and the NHS (54). Among remaining female participants of premenopausal age (*n* = 3; 28.3 ± 6.5 years) and all male participants (*n* = 10), the pomegranate intervention reduced TMAO AUC by 15% compared with placebo (placebo/pomegranate ratio = 1.15; 95% CI: 1.01, 1.31; *P* = 0.036), as estimated from linear mixed models on log-transformed AUC values (Figures 6C, D). On a linear scale, this corresponds to mean TMAO AUC of 1,039 μmol/L × h for placebo and 904 μmol/L × h for pomegranate intervention.

#### Pharmacokinetic parameters for plasma TMAO, L-carnitine, and TMA

Pharmacokinetic parameters for plasma TMAO, L-carnitine, and TMA are presented in Table 3. Only trace quantities of γ-BB were detected in plasma, consistent with literature reports (55, 56). Therefore, γ-BB parameters are not included in the table. No significant differences were observed between placebo and pomegranate conditions for TMAO C_*max*_, T_*max*_, or t_1/2_ (Supplementary Figure 8C). Previous studies investigating TMAO responses after an OCC did not measure TMAO between 8 and 24 h and assumed T_*max*_ occurred at 24 h (22, 33). We observed that T_*max*_ occurred before 24 h for pomegranate intervention, with a mean of 21.6 ± 3.0 h (Table 3). For placebo intervention, mean T_*max*_ was 25.8 ± 11.5 h but varied substantially across individuals, demonstrating the importance of sampling before 24 h to capture complete pharmacokinetic profiles. TMAO t_1/2_ estimates derived from log-linear elimination phase starting after T_*max*_ were 7.8 ± 1.5 h and 8.6 ± 3.4 h for placebo and pomegranate interventions, respectively, indicating relatively efficient elimination after peak TMAO concentrations.

**TABLE 3:** Pharmacokinetic parameters of plasma TMAO, L-carnitine, and TMA.

Analyte	Parameter	Placebo (mean ± SD)	Pomegranate (mean ± SD)	*P*-value
TMAO	AUC (μmol/L × h)^1^	1,096 ± 348	1,161 ± 542	0.897
	C_*max*_ (μmol/L)	43.7 ± 11.9	48.7 ± 23.6	0.737
	T_*max*_ (h)	25.8 ± 11.5	21.6 ± 3.0	0.676
	t_1/2_ (h)^2^	7.8 ± 1.5	8.6 ± 3.4	0.367
L-carnitine	AUC (μmol/L × h)	1,771 ± 629	1,836 ± 482	0.737
	C_*max*_ (μmol/L)	41.3 ± 13.6	42.5 ± 11.2	0.660
	T_*max*_ (h)	20.4 ± 8.8	19.6 ± 2.1	0.715
	t_1/2_ (h)	ND^3^	ND^3^	ND^3^
TMA	AUC (μmol/L × h)	19.2 ± 8.9	17.7 ± 6.9	1.000
	C_*max*_ (μmol/L)	0.8 ± 0.4	0.7 ± 0.2	0.409
	T_*max*_ (h)	7.8 ± 9.7	8.2 ± 13.5	1.000
	t_1/2_ (h)	53.3 ± 36.7	37.8 ± 23.3	0.554

^1^AUC values shown in this table were calculated directly from each participant’s concentration-time data using noncompartmental methods, and therefore differ from the model-based estimated mean AUCs reported elsewhere in the manuscript, which were obtained from linear mixed-effects models fitted on log-transformed AUC and back-transformed to the original scale (estimated marginal means).

^2^Negative TMAO t_1/2_ values excluded; mean ± SEM values obtained for 14 participants.

^3^L-carnitine t_1/2_ could not be determined as C_*max*_ was not captured. ND, not determined; AUC, area under the curve; C_*max*_, maximum concentration; T_*max*_, time to maximum concentration; t_1/2_, half-life. Pharmacokinetic parameters were calculated descriptively for each participant using noncompartmental methods, with area under the curve (AUC) derived by the trapezoidal rule. Values are presented as arithmetic mean ± SD for all participants (*n* = 16). *P*-values were derived from paired comparisons between placebo and pomegranate interventions using paired *t*-tests for AUC and Wilcoxon signed-rank tests for all other pharmacokinetic parameters.

L-carnitine pharmacokinetic parameters showed C_*max*_ below expected values for a 1.5 g dose. Consequently, the T_*max*_ that was derived based on the time point corresponding to the highest observed L-carnitine concentration is approximately 20 h. This is unlikely considering that it exceeds previously reported values of 2–4.5 h (57). T_*max*_ may have occurred between 2.5 and 16 h, suggesting the pharmacokinetic curve did not capture true C_*max*_ (Supplementary Figure 8A). This also precluded accurate t_1/2_ determination, indicated as “not determined” in Table 3. Plasma TMA concentrations were relatively low, averaging approximately 0.4 μmol/L (Supplementary Figure 8B), with C_*max*_ of 0.7–0.8 μmol/L at approximately 8 h (Table 3). This aligns with previous reports that 95% of TMA undergoes oxidation to TMAO (58), indicating highly efficient hepatic conversion.

#### Urolithin production and metabotypes

The presence of urolithins in the urine confirmed that the pomegranate extract reached the colon and underwent microbial metabolism to ellagic acid metabolites (Supplementary Figures 1, 2). To explore potential associations between urolithin metabotype and TMAO response variability, plasma TMAO AUC was stratified by metabotype. Among 16 participants completing Phase II, 3 (18.8%) produced no urolithins (UM-0), 5 (31.3%) produced both urolithins A and B (UM-B), and 8 (50%) produced only urolithin A (UM-A). This distribution largely corresponds to reported literature prevalence of 25%–80% for UM-A, 10%–50% for UM-B, and 10%–25% for UM-0 (59, 60). No significant differences were observed across metabotypes in TMAO AUC change between placebo and pomegranate interventions (Supplementary Figure 3), but mean TMAO AUC values differed between metabotypes following pomegranate intervention (Supplementary Figure 4).

#### Association between TMAO response and kidney function

Previous reports have provided evidence that elevated plasma TMAO is associated with impaired kidney function (61). Here, we observed that serum creatinine was positively associated with TMAO AUC difference between placebo and pomegranate interventions (β = 26.0, 95% CI: 11.4, 40.6; *P* = 0.002), indicating a more favorable pomegranate extract effect on reducing TMAO with poorer kidney function (Supplementary Figure 5). After adjusting for age and sex as potential confounders, the association between creatinine and AUC difference remained significant (β = 20.8, 95% CI: 0.7, 40.8; *P* = 0.045).

## Discussion

This study investigated the effects of a polyphenol-rich pomegranate extract on plasma TMAO concentrations in healthy, omnivorous adults following an OCC. To our knowledge, this is the first study of the effects of a polyphenol-rich extract on the TMAO response to an OCC. In the complete study population (*n* = 16), there was no evidence that pomegranate extract prevented plasma TMAO production.

When “Older” female participants (*n* = 3; 53.3 ± 5.7 years) were excluded (remaining *n* = 13), there was a borderline statistically significant effect, with pomegranate extract reducing TMAO log AUC by 15% compared with placebo. A significant age group × sex interaction on the effect of pomegranate was observed, but is based on an extremely small number of individuals so should be treated with caution.

Ninety one percent of participants meeting our Phase I inclusion criteria produced substantial TMAO quantities from L-carnitine. This proportion substantially exceeds that reported by Wu et al. (33), who classified 45% of participants as high-TMAO producers using a threshold of ≥ 10 μmol/L plasma TMAO at 24 h following a 1.5 g OCC. Although our classification criteria were based on the TMAO increase from baseline to 24 h (> 5 μmol/L and > 50%) rather than absolute 24-h concentrations, a *post hoc* analysis of the TESSA study population demonstrated that 87.5% of participants (28 of 32) exceeded 10 μmol/L at 24 h, qualifying as high-TMAO producers by Wu et al. (33) criteria. The higher proportion of high producers in our study likely reflects our recruitment of omnivores consuming ≥ 4 meat portions weekly, whereas Wu et al.(33) included both omnivores and vegetarians.

Phase I data demonstrated that older age is correlated with a greater TMAO response from baseline to 24 h. These observations are consistent with a crossover study in 20 adults reporting significantly higher fasting plasma TMAO in participants above median age (45.5 years) (56). Another investigation showed elevated plasma TMAO in older (64 ± 7 years) versus younger (22 ± 2 years) adults (62). Serum TMAO concentrations also increased with age in a longitudinal prospective cohort study following 592 healthy participants from age 11 to 26 years (63).

Phase II data revealed an age group × sex interaction on the intervention effects, indicating that the effect of the pomegranate extract on TMAO reduction varied by the combination of age and sex. Female participants in the “Older” group (*n* = 3; 53.3 ± 5.7 years), defined by median split, did not exhibit plasma TMAO AUC reduction following pomegranate intervention. But, their TMAO AUC increased compared with placebo, representing a directionally opposite response to that observed in female participants of premenopausal age and male participants. Although only three of 16 participants were female participants in the “Older” (postmenopausal age) group, existing literature indicates that gut microbiome characteristics differ substantially between sexes, with women typically exhibiting greater microbial richness than men (64, 65). Evidence suggests that the pattern of higher gut microbiome diversity in women compared with men during younger years may not persist into older adulthood (65), indicating that older women potentially exhibit microbiome profiles more similar to men. This hypothesis is supported by a study demonstrating compositional gut microbiome differences between premenopausal women (*n* = 44) and men (*n* = 42) that were considerably less pronounced between postmenopausal women (*n* = 45) and men (*n* = 48) (66). These observations may therefore reflect distinct microbiome profiles in women of postmenopausal age compared with men and women of premenopausal age. Future studies should investigate associations between TMAO responses and specific microbial taxa. During Phase II, participants collected fecal samples before and after each OCC. These samples can be analyzed for L-carnitine- and γ-BB-metabolizing gene abundance (*cai* and *gbu* gene clusters), such that the change in abundance before and after the OCC can be compared between the pomegranate and placebo interventions.

### Study limitations

Although pomegranate extract effects on TMAO production were observed in female participants of premenopausal age and male participants, this remains a proof-of-concept study with a small population and smaller subgroups, including only three female participants in the “Older” (postmenopausal age) group and three in the “Younger” (premenopausal age) group. Since the study was not powered for age group and sex stratification, data interpretation requires caution.

Another limitation involves difficulty establishing L-carnitine pharmacokinetic parameters. For instance, t_1/2_ values could not be derived because plasma L-carnitine concentrations remained relatively stable over 48 h. Since only a small proportion of supplemental L-carnitine is absorbed compared with dietary L-carnitine, with reported bioavailability of only 14%–18% (31), complete L-carnitine absorption into blood was unlikely. Yet if 18% of 1.5 g L-carnitine were absorbed (equivalent to 270 mg L-carnitine), approximately 335 μmol/L plasma L-carnitine increase would be expected, whereas mean peak concentration observed was only 42 μmol/L. Previous absorption, distribution, metabolism, and excretion studies showed L-carnitine T_*max*_ occurred at 2–4.5 h post-administration (57). Therefore, L-carnitine concentrations may have reached T_*max*_ after 2.5 h (and before 16 h), which was not captured and may have affected t_1/2_ determination. To better understand L-carnitine supplement absorption, distribution, metabolism, and excretion, collected urine samples should be analyzed for L-carnitine, TMAO, and other methylamine presence.

A further mechanistic consideration is whether the pomegranate extract may have influenced plasma TMAO concentrations partly through its effect on the absorption of L-carnitine in the small intestine, rather than solely through inhibition of microbial TMA production. In Phase II, we observed a non-significant trend toward higher plasma L-carnitine concentrations in the pomegranate extract condition compared to placebo across the 48-h pharmacokinetic period (Supplementary Figure 8A). If the pomegranate extract increased intestinal absorption of L-carnitine, a greater proportion of the ingested dose would be taken up before reaching the colon, theoretically reducing the substrate available to TMA-producing gut microbiota and thereby lowering TMAO production through a mechanism independent of direct microbial inhibition. The bioavailability of L-carnitine from supplements is between 14%–18% (31), with the remainder passing into the colon where gut microbiota convert it to TMA. Intestinal L-carnitine absorption occurs primarily via the high-affinity transporter OCTN2, with a potential additional contribution from the low-affinity ATB0,+ transporter, especially under conditions of high supplemental L-carnitine intake (67). Whether pomegranate polyphenols could modulate the activity or expression of these transporters is currently unknown. Previous studies have reported that polyphenols extracted from sweet cherry interact directly with the mitochondrial carnitine/acylcarnitine carrier (CACT) in a molecular docking model, with binding at the transporter’s active site (68). This raises the possibility that structurally related polyphenols from pomegranate could also interact with intestinal carnitine transport proteins, but this remains speculative. Taken together, while the observed trend toward higher circulating L-carnitine in the pomegranate condition warrants further investigation, the data presented here do not allow us to distinguish between enhanced absorption versus delayed microbial catabolism as contributing factors.

## Conclusion

In healthy, omnivorous adults (*n* = 16), a single dose of pomegranate extract (1.6 g) did not reduce plasma TMAO response following an OCC. However, the extract produced a significant 15% reduction in TMAO response in female participants of premenopausal age and male participants (*n* = 13), indicating potential sex-dependent cardiovascular benefits from polyphenol interventions targeting the gut microbiome-TMAO pathway. A significant age group × sex interaction was observed, with pomegranate extract reducing plasma TMAO AUC in female participants of premenopausal age and male participants while showing opposite effects in female participants assigned to the “Older” (postmenopausal age) group. These findings suggest that dietary polyphenol strategies to reduce TMAO may require sex- and age-specific considerations for cardiovascular disease prevention.

## Data Availability

The datasets presented in this study can be found in online repositories. The names of the repository/repositories and accession number(s) can be found below: the anonymised data that support the findings of this study are openly available in Zenodo at 10.5281/zenodo.18457092.
