# Supplementary material for: Effect of a polyphenol-rich pomegranate extract on plasma trimethylamine N-oxide levels following an oral carnitine challenge: a randomized controlled crossover trial in healthy adults
Source: Front Nutr. 2026 May 20;13:1822840. doi: 10.3389/fnut.2026.1822840 (PMC13260717; doi:10.3389/fnut.2026.1822840)
Supplement: Supplementary file 1 [file Data_Sheet_1.pdf]

Supplementary information

Supplementary methods

Urolithin relative response factors

Commercially available urolithin standards are aglycones; however, urinary urolithins predominantly exist as glucuronide or sulfate conjugates. Therefore, enzymatic hydrolysis was performed on one urine sample using glucuronidases and sulfatases to determine the response ratio between aglycone and conjugated forms. A 24-hour urine collection from an individual who received pomegranate intervention was divided into ten 200-µL aliquots. Standard curves were prepared in baseline urine (presumed free of pomegranate compounds) divided into five 200-µL aliquots with increasing urolithin A and B aglycone concentrations up to 16 µg/mL. Ten microliters taxifolin (20 µg/mL) and 520 µL phosphate buffer (pH 6.8) were added to each aliquot. Half the samples and all standards received 80 µL glucuronidase (1000 U/mL) and 80 µL sulfatase (1000 U/mL); remaining samples remained untreated (non-hydrolyzed). All aliquots were incubated at 37°C for 2 hours. Hydrolysis was terminated by adding 570 µL dimethylformamide and 40 µL formic acid. Samples were vortexed, centrifuged at maximum speed for 2 minutes, and 400 µL was filtered through 0.45-µm Whatman Mini-UniPrep filters (Cytiva, Marlborough, MA, USA) before LC-MS/MS analysis.

Sulfate conjugates were not detected; therefore, only glucuronide-to-aglycone ratios were established. Urolithin concentrations in the 24-hour collection were calculated by multiplying quantities by total urine volume and converting to µmol/L. Non-hydrolyzed samples contained mean concentrations of 2.5 µmol/L urolithin A and 0.6 µmol/L urolithin B (**Supplementary Figure S1A**), whereas hydrolyzed samples contained 30.8 µmol/L urolithin A and 15.3 µmol/L urolithin B (**Supplementary Figure S1B**). Relative response factors were calculated as:

$$RRF = \frac{C\ aglycone, hydrolysed - C\ aglycone, non\ hydrolysed}{C\ glucuronide, non\ hydrolysed}$$

Mean relative response factors were 2.13 for urolithin A glucuronide to urolithin A and 2.75 for urolithin B glucuronide to urolithin B.

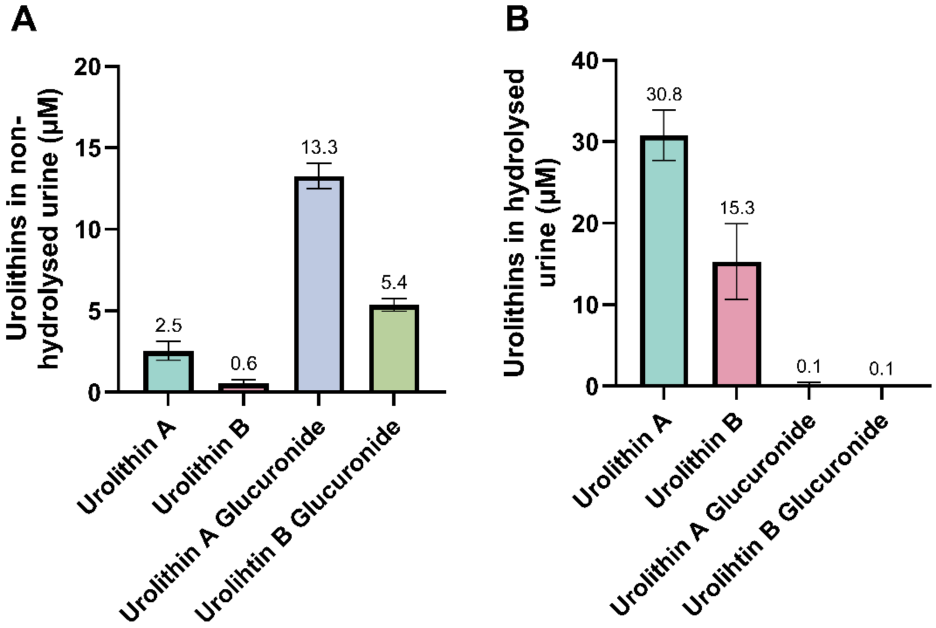

Supplementary Figure S1. Urolithin concentrations in a 24-hour urine collection after the consumption of a pomegranate extract. Part of the urine was (**A**) left untreated, while another part was (**B**) enzymatically hydrolyzed, such that the conversion of urolithin conjugates into their aglycone forms could be determined. Five aliquots were incubated with glucuronidase/sulfatase (1000 U/mL), while five were kept untreated. Samples were incubated at 37 °C for 2 h, after which hydrolysis was halted with dimethylformamide (DMF). Urolithins were quantified by LC-MS/MS using urolithin A and B standards.

Statistical analyses

To test for kidney function interactions, linear regression models were fitted separately for each intervention arm (placebo, pomegranate) and for the difference between arms (placebo minus pomegranate). Creatinine concentrations measured during Phase I served as kidney function markers. The eGFR marker was not used because it had a ceiling value of 90 mL/min/1.73 m^2,

which most participants exceeded. Each model included creatinine as primary predictor with age and sex as covariates. Interaction terms between sex and creatinine were tested to assess sex-specific associations.

For each participant, the coefficient of variation of baseline plasma TMAO was calculated across three time points (two for participants with incomplete data): baseline measurements during Phase I, Phase II intervention 1, and Phase II intervention 2 (time point 0). Coefficient of variation values assessed intra-individual variability in baseline plasma TMAO across study phases.

Supplementary Figures

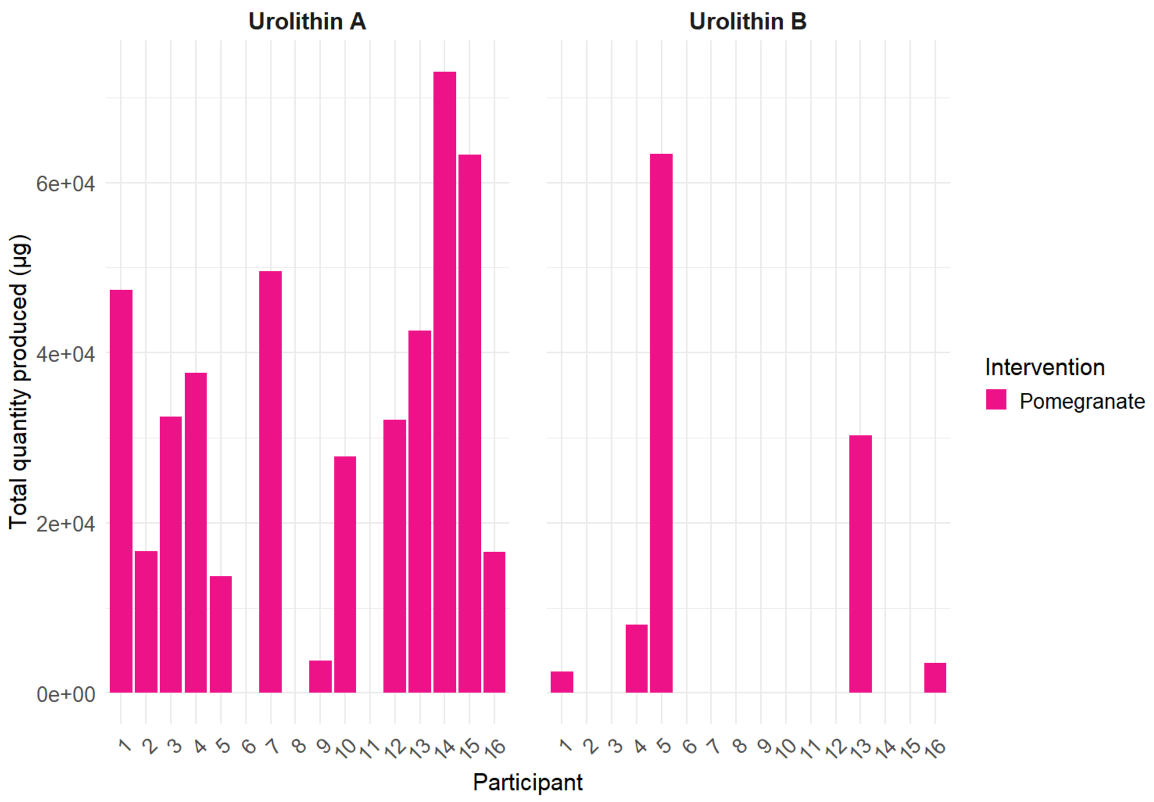

Supplementary Figure S2. Total urolithin production in TESSA participants following the consumption of 1.6 g pomegranate extract. For each participant (n=16), accumulated urolithin production over 48 hours was calculated by multiplying measured urolithin concentrations in urine aliquots by the corresponding total urine volume. Glucuronidated urolithins were converted to aglycone equivalents using the established relative response factor (RRF; see Supplementary Figure S1). All urine collected during 48 hours was stored at -80 °C before LC-MS/MS analysis. Participant numbers reflect rank-ordering from greatest to smallest change in the TMAO AUC induced by the pomegranate extract relative to placebo.

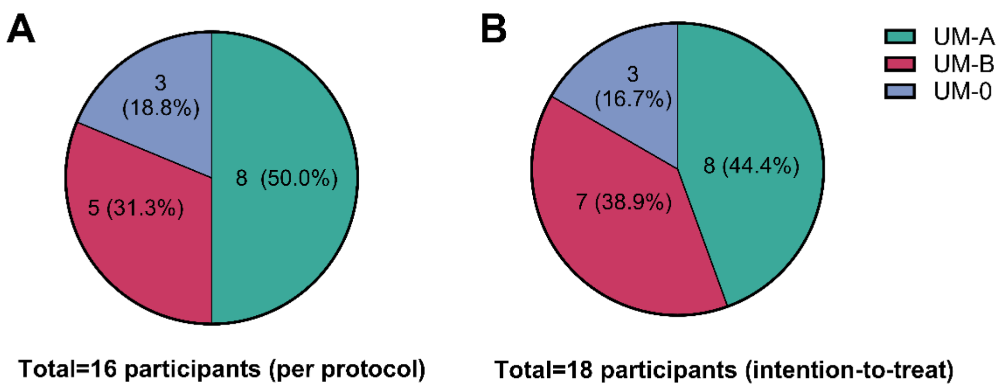

Supplementary Figure S3. Distribution of urolithin metabotypes among TESSA participants. Urolithin profiles obtained after pomegranate-extract supplementation were used to assign participants to metabotypes UM-A (urolithin A only), UM-B (urolithin A and B), or UM-0 (no detectable urolithins), using a threshold of 0.3 ppm. Data are shown for (**A**) participants who completed Phase II per protocol (n=16) and (**B**) all individuals who completed the pomegranate intervention (intention-to-treat, n=18).

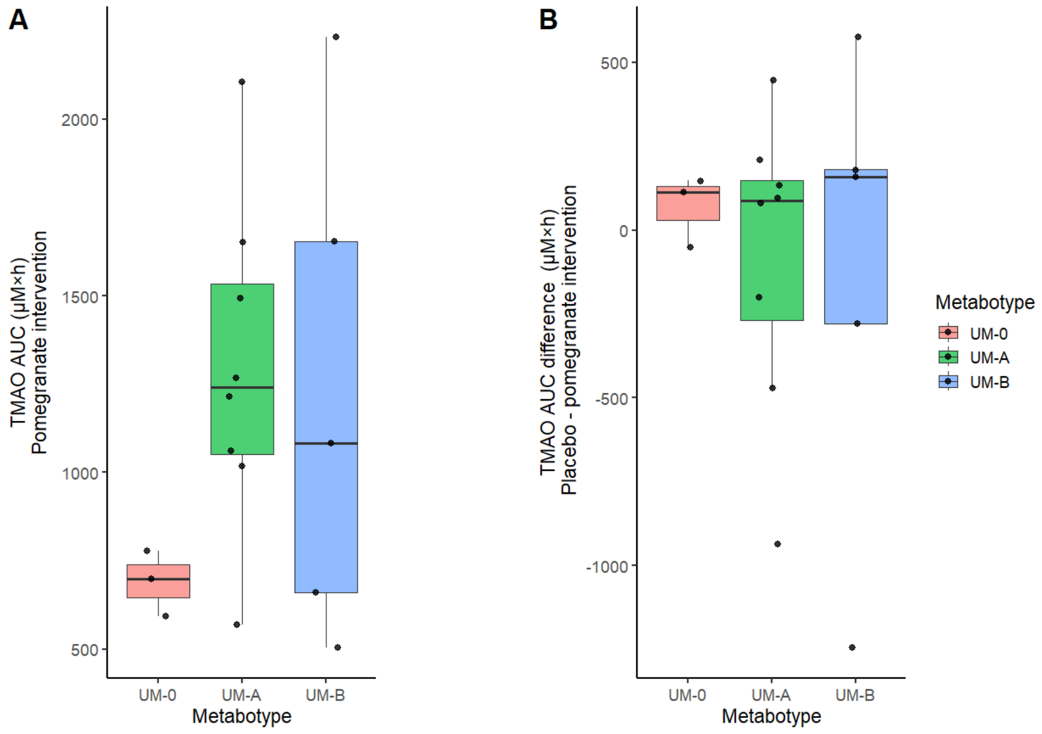

Supplementary Figure S4. Plasma trimethylamine N-oxide responses across urolithin metabotypes. (**A**) Plasma TMAO area under the curve (AUC) following the pomegranate intervention and (**B**) the within-participant difference between placebo and pomegranate AUC values are displayed for each urolithin metabotype: UM-0 (n=3), UM-A (n=8), and UM-B (n=5).

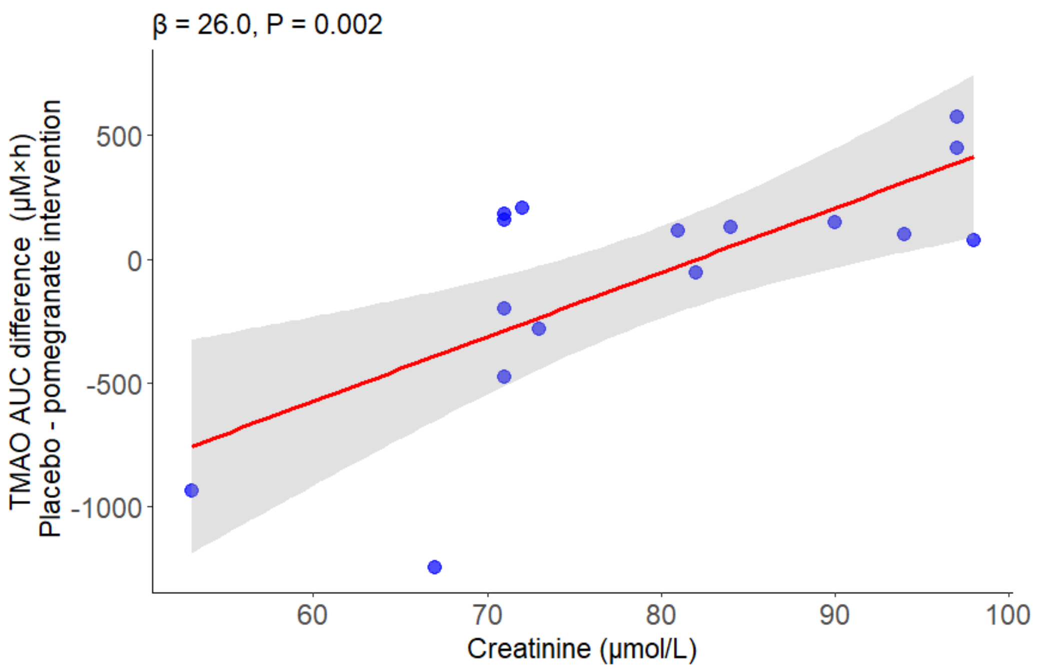

Supplementary Figure S5. Association between serum creatinine and the effect of the pomegranate extract on plasma trimethylamine N-oxide area under the curve (TMAO AUC). Higher baseline serum creatinine concentrations were associated with smaller reductions in TMAO AUC following the pomegranate extract relative to placebo. Individual values are shown (n=16). Serum creatinine was measured during Phase I and TMAO AUC was derived from blood plasma samples collected over 48 hours during Phase II after administration of a 1.5 g oral carnitine challenge (OCC).

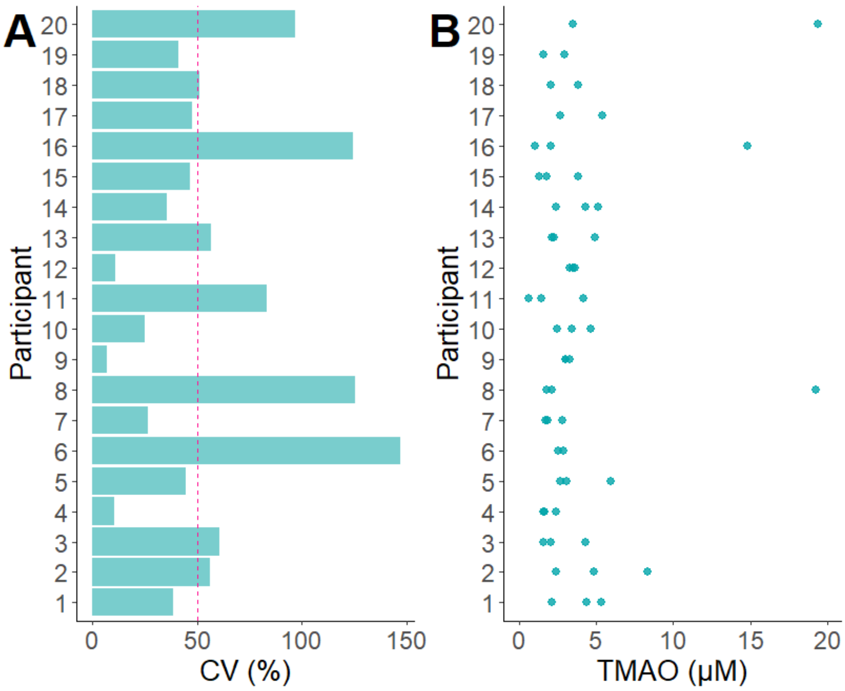

Supplementary Figure S6. Variation in fasting baseline plasma trimethylamine N-oxide (TMAO) concentrations within participants measured on three occasions (Phase I, Phase II-intervention 1, and Phase II-intervention 2). (**A**) Coefficients of variation and (**B**) individual values are shown for baseline plasma TMAO concentrations for all 20 participants entering Phase II. Most participants had three fasting measurements, while participants 17-20 had two (due to drop-out prior to entering Phase II-intervention 2). Plasma was obtained by centrifuging whole blood samples and stored at −80 °C prior to LC-MS/MS analysis using a d9-TMAO internal standard.

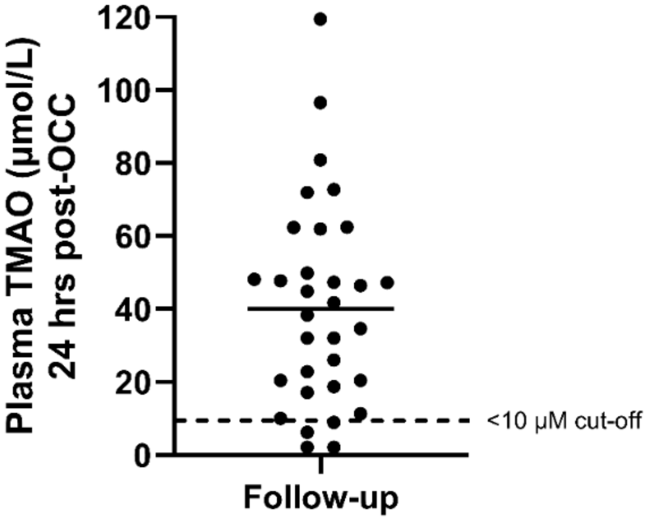

Supplementary Figure S7. Distribution of blood plasma trimethylamine N-oxide (TMAO) levels measured 24 hours after the oral carnitine challenge (OCC) across participants in Phase I, with <10 µM cut-off shown. Data points represent individual Phase I participants (n=32). The cut-off is based on a report by Wu *et al.* (2020) (1) to identify high-TMAO producers. At 24 hours post-OCC, 4 out of 32 participants showed TMAO levels <10 µM. Fasted blood samples were collected from TESSA participants prior to the OCC in Phase I. Whole blood samples were centrifuged to obtain plasma and stored at -80 °C until LC-MS/MS quantification using a d9-TMAO internal standard.

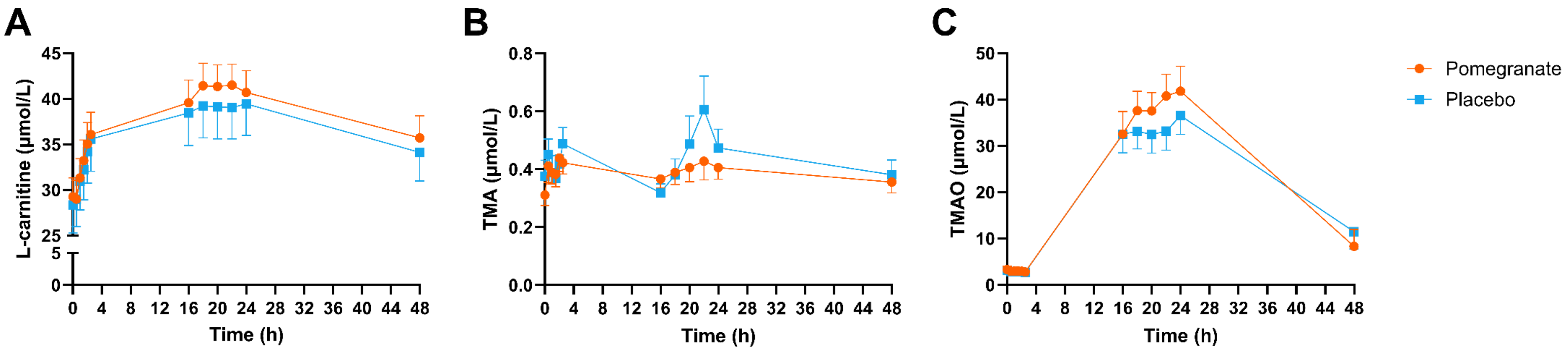

Supplementary Figure S8. Concentrations of (A) L-carnitine, (B) trimethylamine (TMA), and (C) trimethylamine N-oxide (TMAO) over 48 hours measured in blood plasma of TESSA participants who underwent two oral carnitine challenges (OCCs), consuming 1.5 g L-carnitine once with a placebo and once with a pomegranate extract. Data are shown as mean ± SEM across 16-18 participants. Pharmacokinetic parameters reported in Table 3, including T_{max}, were derived from individual observed concentration-time profiles using noncompartmental analysis and may therefore not correspond to the peak of the group mean curve, as a result of inter-individual variability in the time of peak concentration. During 48 hours after the OCC blood plasma was collected to estimate a pharmacokinetic curve for L-carnitine, TMA, and TMAO. Whole blood samples were centrifuged to obtain plasma and stored at -80 °C until LC-MS/MS quantification using isotope-labelled internal standards.

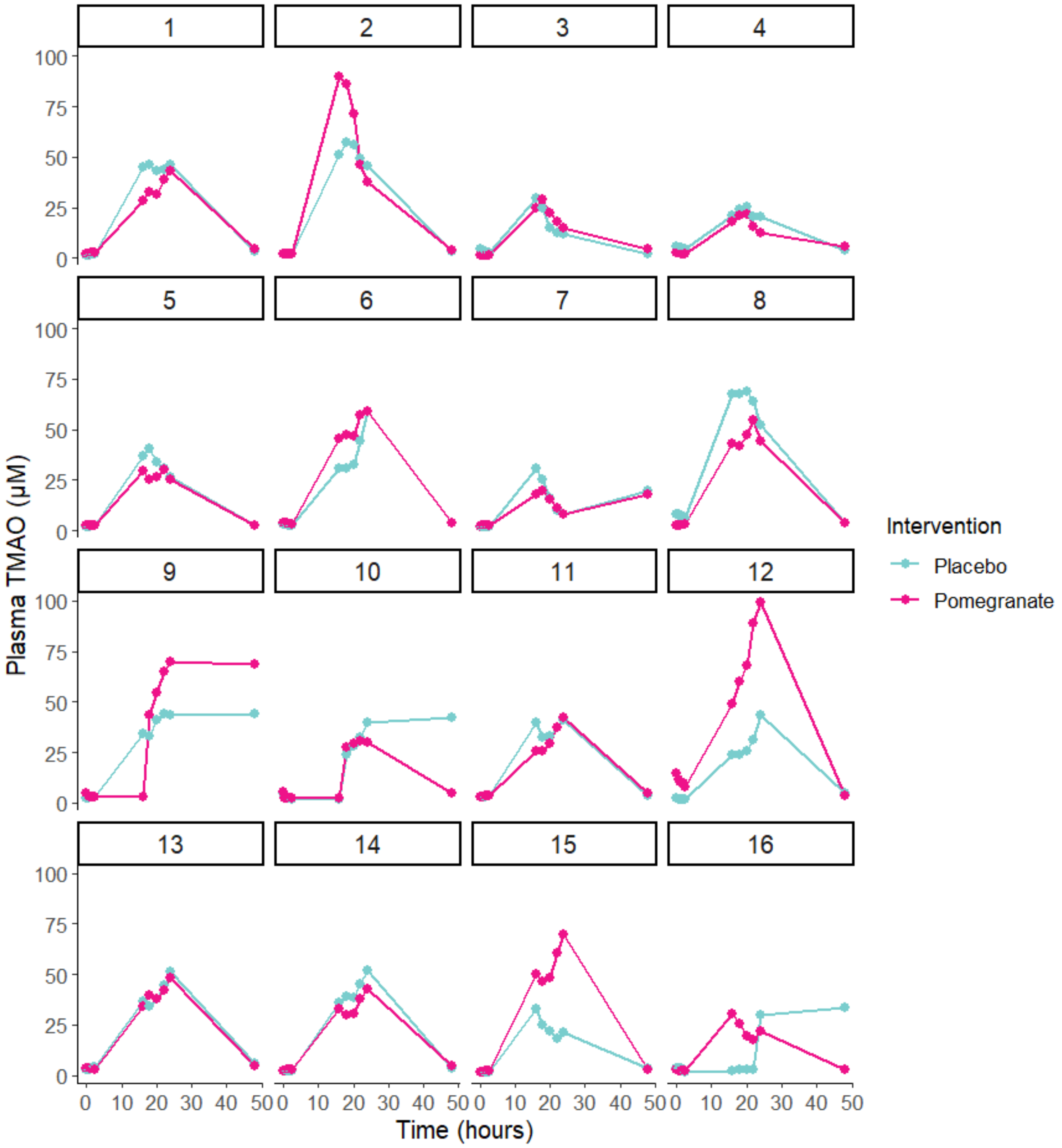

Supplementary Figure S9. Concentrations of individual participants' plasma trimethylamine N-oxide (TMAO) levels over 48 hours. During 48 hours after the OCC blood plasma of 16 participants was collected to estimate the TMAO pharmacokinetic curve for each participant. Whole blood samples were centrifuged to obtain plasma and stored at -80 °C until LC-MS/MS quantification using isotope-labelled internal standards.

References

1. Wu W-K, Panyod S, Liu P-Y, Chen C-C, Kao H-L, Chuang H-L, et al. Characterization of TMAO productivity from carnitine challenge facilitates personalized nutrition and microbiome signatures discovery. Microbiome. 2020;8(1):162.
